# Degree-Strength Correlation Reveals Anomalous Trading Behavior

**DOI:** 10.1371/journal.pone.0045598

**Published:** 2012-10-17

**Authors:** Xiao-Qian Sun, Hua-Wei Shen, Xue-Qi Cheng, Zhao-Yang Wang

**Affiliations:** Institute of Computing Technology, Chinese Academy of Sciences, Beijing, China; Cinvestav-Merida, Mexico

## Abstract

Manipulation is an important issue for both developed and emerging stock markets. Many efforts have been made to detect manipulation in stock markets. However, it is still an open problem to identify the fraudulent traders, especially when they collude with each other. In this paper, we focus on the problem of identifying the anomalous traders using the transaction data of eight manipulated stocks and forty-four non-manipulated stocks during a one-year period. By analyzing the trading networks of stocks, we find that the trading networks of manipulated stocks exhibit significantly higher degree-strength correlation than the trading networks of non-manipulated stocks and the randomized trading networks. We further propose a method to detect anomalous traders of manipulated stocks based on statistical significance analysis of degree-strength correlation. Experimental results demonstrate that our method is effective at distinguishing the manipulated stocks from non-manipulated ones. Our method outperforms the traditional weight-threshold method at identifying the anomalous traders in manipulated stocks. More importantly, our method is difficult to be fooled by colluded traders.

## Introduction

Stock market provides companies with a place to raise money and allows the investors to trade their shares conveniently. Generally speaking, the price of a stock reflects the common judgment to the value of the stock and it is determined by the supply and demand of the stock if without any interference. However, stock prices could be manipulated by spurious information and fraudulent trades. Manipulation of stock prices affects investors' confidence, disturbs the market order and is harmful to the development of stock markets. Therefore, manipulation is an important issue for both developed and emerging stock markets.

The pioneering work of stock price manipulation classified the manipulations into three types: action-based manipulation, information-based manipulation and trade-based manipulation [1], [2]. Earlier studies mainly investigated the action-based and information-based manipulation, such as insider dealing [3], [4]. Recent research interests focused on the trade-based manipulation, which is more common in current stock markets. Güray Küçükkocaoğlu examined the closing-price manipulation in the Istanbul Stock Exchange [5]. Aggarwal and Wu developed a model to explain trade-based manipulation and tested the model using data from US stock markets [6]. Sun et al. investigated the statistical properties of trading activity and pointed out the differences between non-manipulated stocks and manipulated stocks [7].

Recently, the detection of manipulation has attracted much research attention. Most approaches adopted the supervised learning framework in which detection models are trained using the transactions which have been carefully judged as anomalous or normal ones a priori. Palshikar and Bahulkar attempted to identify the pattern of manipulation using fuzzy temporal logic [8]. Öğüt et al. detected stock price manipulation using artificial neural networks and support vector machine [9]. However, the supervised learning framework depends heavily on the quality and size of training data, which is very difficult to obtain in practice. Alternatively, several other methods, such as clustering technique and statistical method, were employed to detect manipulation. Palshikar and Apte applied graph clustering techniques to detect the candidate collusion sets [10]. Sun et al. investigated the detection of trade-based manipulation in Chinese stock markets by analyzing trading networks [11].

Although many efforts have been made to detect manipulation, it is still an open problem to identify the fraudulent traders, especially when they collude with each other. In trade-based manipulation, a group of colluded traders acts together to create an artificial demand for the stock and attracts other investors to buy the stock. In this way, the group of traders can make profit by selling their shares when the stock price rises sufficiently. The manipulation from colluded traders appears frequently in emerging stock markets, such as Chinese stock market, where a single trader can control thousands of accounts. In addition, such type of manipulation usually lasts for a long time. This poses a big challenge to the surveillance systems of exchange, which only analyze short-term transaction data in the limited time.

In this paper, we focus on the problem of identifying the anomalous traders using the transaction data. For each stock, we construct a stock trading network which depicts the frequency of transaction among traders. By analyzing the correlation between degree and strength of nodes in the stock trading network, we find that the non-manipulated stocks behave almost identically to their randomized counterparts while the manipulated stocks perform very differently. Motivated by this finding, we take the randomized network as null model and then identify the anomalous traders by checking the statistical significance of the ratio of strength to degree. We test our method on 44 non-manipulated stocks and 8 manipulated stocks from Chinese stock market. Results demonstrate the effectiveness of our method at distinguishing the manipulated stocks from non-manipulated ones. Moreover, our method outperforms the traditional weight-threshold method at identifying the anomalous traders in manipulated stocks.

## Results

### The stock trading network

Promoted by the success of network theory in many interdisciplinary fields [12]–[16], network has been increasingly used to investigate the financial systems. For example, much research attention has been paid to investigate stock markets from the perspective of complex networks [17]–[25]. In recent years, with the increasing availability of transaction data to researchers, stock trading network is taken as a convenient tool to characterize the trading relationships among traders in stock markets. Similar to complex networks from other fields, stock trading network also possesses a power-law degree distribution, a power-law strength distribution, and a power-law weight distribution [11], [26].

Before proceeding, we first introduce two types of widely-used stock trading networks, namely trading volume network and trading times network. Nodes in the two types of stock trading networks correspond to traders involved in the transactions of stock. An edge connects two traders who appear in a transaction. The weight of edge, however, has different physical meanings for the two types of trading networks. For trading volume networks, the weight of en edge represents the volume of the transactions among traders. For trading times network, the weight of edge describes the number of times two traders appear in the same transaction. In this paper, we focus on the trading times network and hereafter we use trading network to refer to the trading times network for convenience. In addition, we use the daily trading network to refer to the trading network constructed according to the transactions in one transaction day. Accordingly, we use the yearly trading network to refer to the trading network constructed according to the transactions in a whole year.

**Figure 1:**
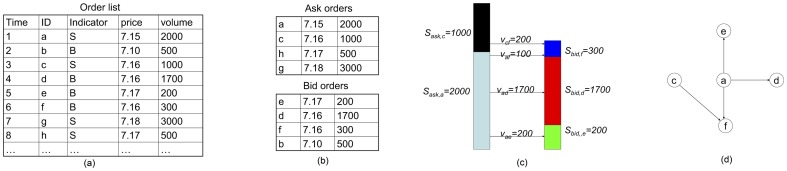
An example of the order matching process by electronic system in stock exchange. (a) Orders submitted by traders. (b) The list of ask orders and bid orders. (c) The match process. (d) The trading network constructed according to the executed transactions.

In general, the properties of trading network depend heavily on the trading rules regulated by the stock exchange. Thus, we also briefly introduce the trading rules in stock market. In stock market, a trader submits bid/ask orders to the electronic trading system when he/she wants to buy/sell shares. In the trading system, the list of bid orders is sorted in descending order of price and the list of ask orders is sorted in ascending order of price. For each of the two lists of orders, two orders with the same price are sorted according to their submission time. The trading system matches a bid order and an ask order from the top of these two lists. Once a bid order is matched by an ask order, a transaction is executed. If the two matched orders do not have the same volume, the unexecuted part of the order with larger volume is taken as a new order added to in the corresponding list. As an example, Figure 1 gives an illustration of the trading rule in stock market.

### Correlation between strength and degree in trading network

**Figure 2:**
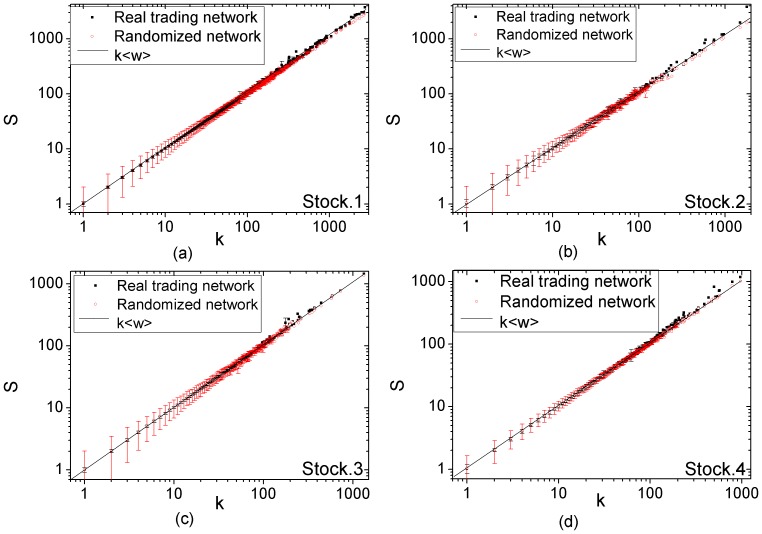
The degree-strength correlation for four randomly-selected non-manipulated stocks. The horizontal axis is the degree *k* of nodes and the vertical axis is the average strength *s*. The solid line is the power-law fits to the function 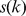 for real trading networks, and the dash line is the power-law fit to the function 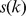 for randomized trading networks.

As illustrated in the previous section, the orders are automatically matched by electronic systems in Stock Exchange. The buyers or sellers cannot choose the transaction counterparts by themselves. This indicates that a pair of traders has little chance to trade frequently between them. Therefore, for the trading networks of non-manipulated stocks, it is normal to observe the absence of the correlation between the weight of edge and the degree of the two end nodes, i.e., the strength of a node is simply proportional to its degree. Thus, in terms of the correlation between node strength and node degree, the trading networks should exhibit similar behavior to its randomized counterpart, in which the topology of trading network is retained while the weights of edges are redistributed randomly. Figure 2 shows the correlation between node strength and node degree in the trading networks of 4 randomly-selected non-manipulated stocks. We can see that the average strength 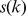 of nodes with degree 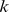 is a linear function of node degree 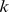. This function can be well fitted by 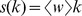, which verifies that traders occurring in the same transaction are matched by chance. In addition, such a phenomenon is almost identical to the randomized version of the stock trading network. This finding clearly demonstrates the absence of structural regularities in the non-manipulated trading networks.

**Figure 3:**
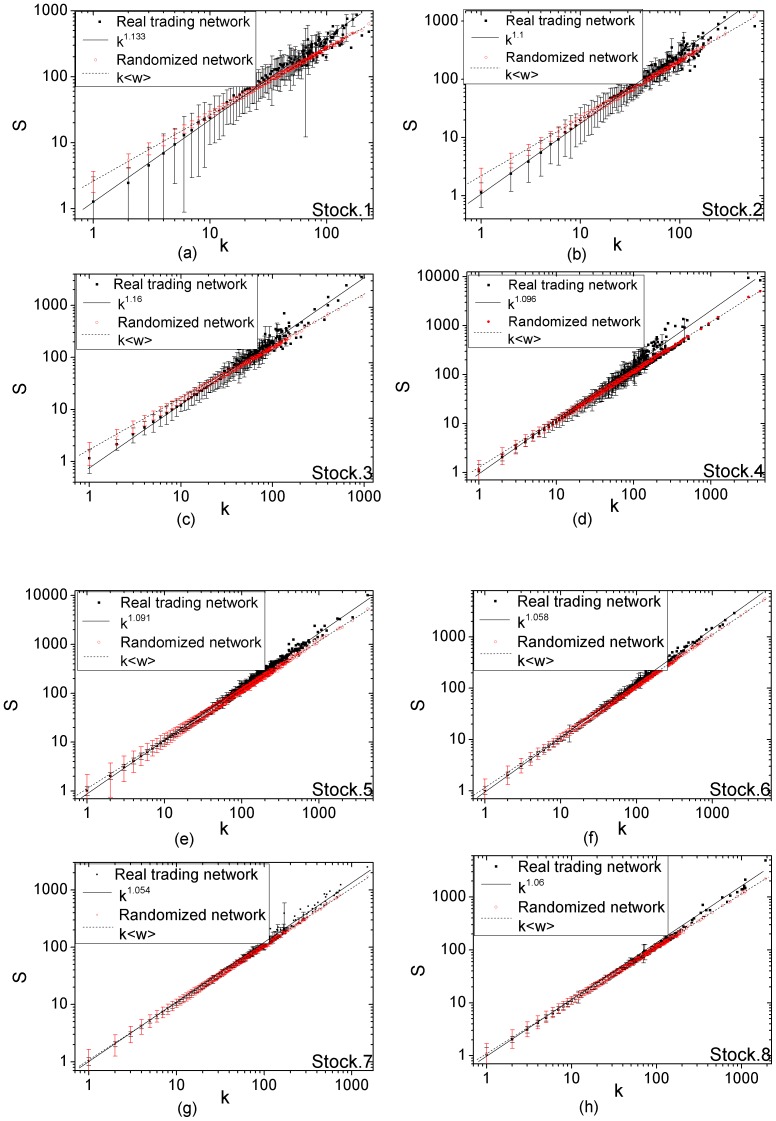
The degree-strength correlation for eight manipulated stocks. The horizontal axis is the degree *k* of nodes and the vertical axis is the average strength *s*. The solid line is the best power-law fit to the function 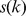 for real trading networks, and the dash line is the power-law fit to the function 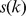 for randomized trading networks. (a)
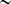
(d) The degree-strength correlation in the trading networks of the stocks which are manipulated for the whole year. (e)
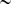
(h) The degree-strength correlation in the trading networks of the stocks which are manipulated from Jan 2004 to Sep 2004.

We further compare the trading network of manipulated stock with its randomized counterpart. As shown in Figure 3, from the perspective of the correlation between the average strength 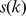 and the degree 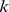, the randomized trading networks for manipulated stocks are the same to the randomized networks for non-manipulated ones. However, the trading networks of manipulated stocks show very different behavior. The average strength 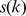 of nodes with degree 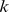 increases with the node degree 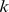 as power functions 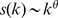 with the exponent 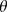 larger than 1. Figure 3 (a)
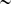
(d) illustrate the correlation of average strength and degree for four manipulated stocks whose manipulation periods last for the whole year. The power-law fit for their trading networks gives anomalous exponent 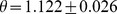. Figure 3 (e)
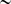
(h) depict the correlation of average strength and degree for the other four manipulated stocks whose manipulation periods end in the year. The power-law exponents are 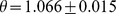. The smaller exponents are attributed to that the manipulators have begun to sell their shares and thus the transactions take place between the manipulators and other traders rather than among the manipulators.

We have shown that the manipulated stocks exhibit higher exponents of the power-law degree-strength correlation function. This implies that the weight of edges depend on the degree of nodes to some extent. One possible explanation is that anomalous traders trade more often among themselves to influence the volume and the price of the target stock. More importantly, the higher edge weight can be attributed to the colluded traders with low degree. These traders deceive the electronic systems in Stock Exchange and form intentional trades among themselves. Their trading behavior may give rise to nonlinear degree-strength correlation among traders with low degree. In sum, the nonlinear degree-strength correlation in manipulated stock networks provides us important clues to identify the anomalous traders and motivates us to use the ratio of strength to degree to identify these anomalous traders in manipulated stocks.

### Identifying anomalous traders by statistical significance

Motivated by correlation between node strength and node degree, we propose a method to use the ratio of strength to degree to identify the anomalous traders in manipulated stocks. We take the traders with higher ratio of strength to degree as candidate anomalous traders. However, one prominent problem is the choice of an appropriate threshold. We adopt the statistical significance to address this problem. Specifically, we take the trader as a candidate anomalous trader if its ratio of strength to degree is significantly higher than expected value.

**Figure 4:**
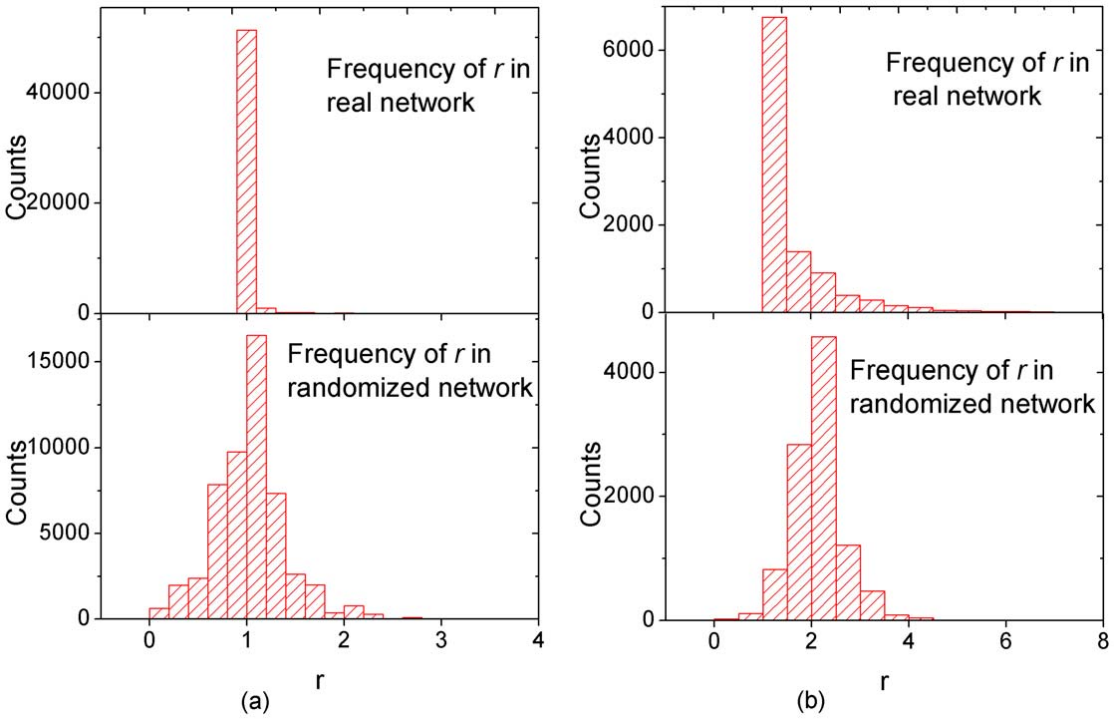
Histogram of 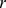
 **for (a)one non-manipulated stock and (b)one manipulated stock.** Distributions of 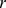 for the real trading network and the randomized network are presented in the top panel and the bottom panel respectively.

To use the method of statistical significance, we should first choose an appropriate null mode for each trading network. Here, the adopted null model is a randomized network with no correlation between node strength and node degree. The randomized network is obtained by retaining the topology of the original trading network and redistributing the weights of edges randomly. For convenience, for node 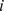 with degree 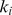 and strength 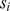, we use 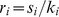 to denote its ratio of strength to degree. Figure 4 shows the distribution of 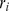 for two trading networks and their randomized counterparts. One trading network is the network for a non-manipulated stock and the other is for a manipulated stock. From Figure 4, we can see that the ratio of strength to degree roughly follows a normal distribution for the two randomized networks. The average 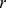 of 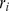 for the randomized networks is 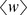 and the standard deviation 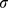 can be easily obtained. For a node 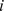 in a trading network, we can compute the z-score as 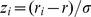. The node 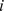 is viewed as candidate anomalous trader if its z-score is larger than 3, which corresponds to the statistical significance level 0.001.

**Figure 5:**
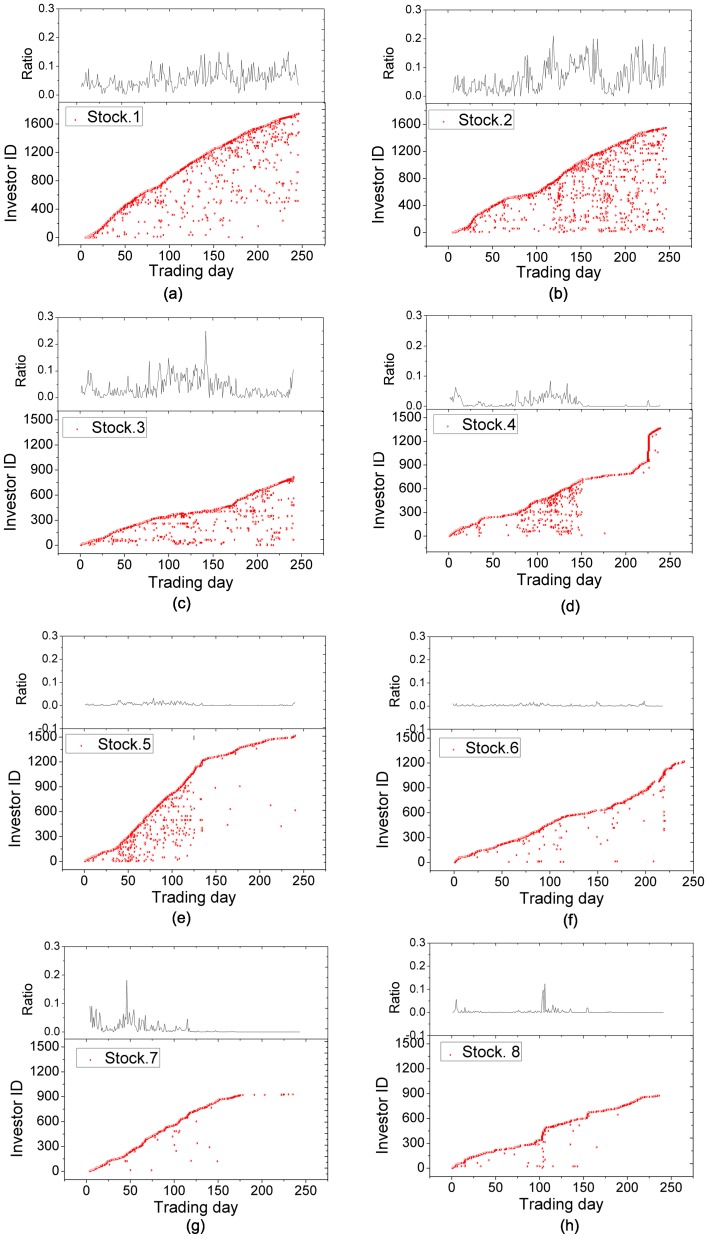
Anomalous traders detected in the trading networks of eight manipulated stocks. The horizontal axis represents trading days and the vertical axis represents these investors's ID reordered according to their appearance in transaction data of the stock. The percentage of anomalous traders is showed in top panels. (a)
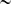
(d): stocks with the manipulation periods persisting the whole year.(e)
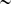
(h): stocks with the manipulation periods from Jan 2004 to Sep 2004.

**Figure 6:**
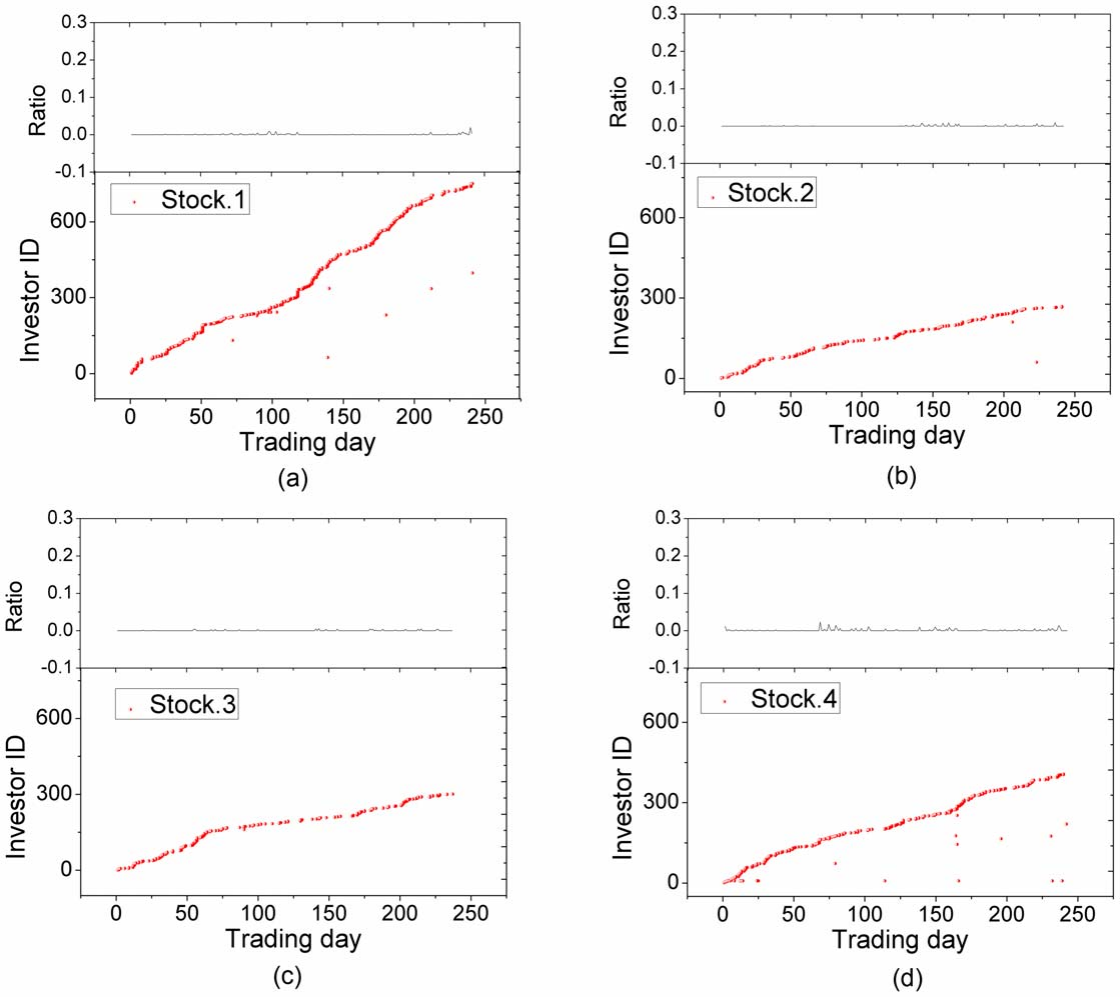
Anomalous traders detected in the trading networks of four randomly-selected non-manipulated stocks. The horizontal axis represents trading days and the vertical axis represents these investors's ID reordered according to their appearance in transaction data of the stock. The percentage of anomalous traders is showed in top panels.

For a stock, we construct the daily trading network for each trading day and use the above proposed method to identify the candidate anomalous traders. Figure 5 shows the detected candidate anomalous traders in the daily trading networks for 8 manipulated stocks. Figure 6 shows the detected candidate anomalous traders in the daily trading networks for 4 randomly-selected non-manipulated stocks. Comparing Figure 5 with Figure 6, we can see that most of the candidate anomalous traders occur in many trading days for manipulated stocks while each of the candidate anomalous traders occurs in very few days for non-manipulated stocks. This demonstrates that the proposed method not only can effectively distinguish the manipulated stocks from the non-manipulated ones, but also can identify the anomalous traders accurately by statistical significance.

Furthermore, our method provides a convenient way to judge the manipulation period of the manipulated stocks. Figure 5 (a)
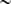
(d) show the obtained candidate anomalous traders in four manipulated stocks whose manipulation periods last the whole year 2004. Figure 5 (e)
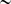
(h) show the obtained candidate anomalous traders in four stocks whose manipulation periods end in the year 2004. We can see that the number of candidate anomalous traders declined sharply when the manipulation periods end. Thus, the sharp decrease provides a clue to determine the end of the manipulation period. Table 1 shows the manipulation periods claimed by China Securities Regulatory Commission (CSRC) and the manipulation periods obtained by our method.

**Table 1:** The manipulation period of eight manipulated stocks.

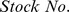	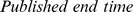	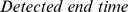
1	2005-12-30	-
2	2005-01-31	-
3	2005-12-30	-
4	2005-12-30	2004-08-20
5	2004-09-20	2004-07-29
6	2004-12-31	2004-12-07
7	2004-09-20	2004-09-20
8	2004-09-03	2004-08-26

**Figure 7:**
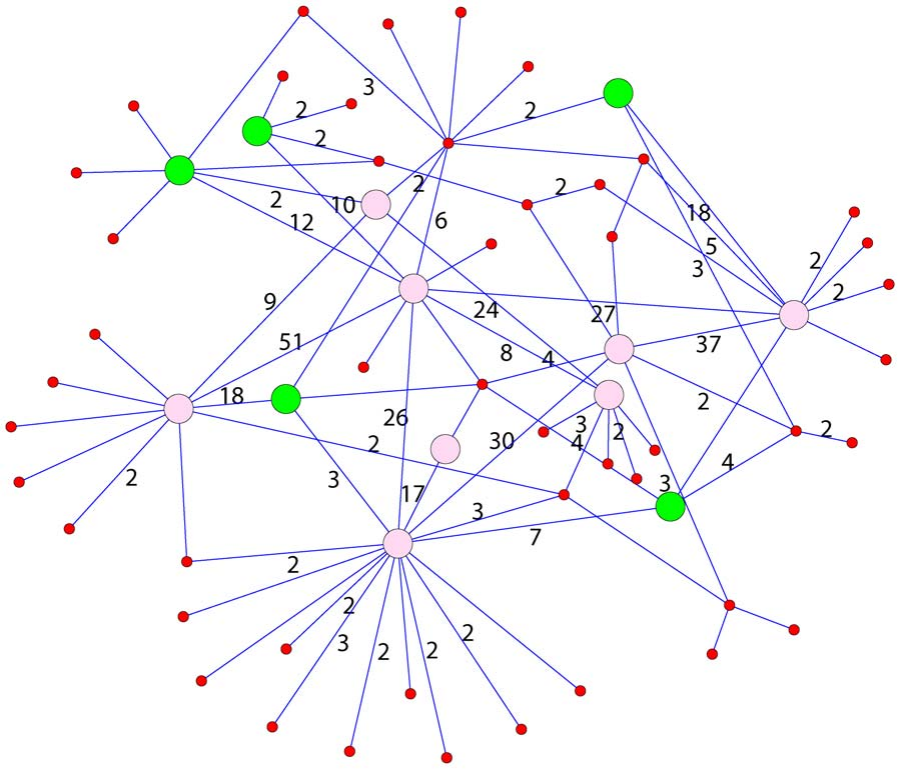
Part of a trading network for a real manipulated stock on one trading day. The bigger nodes are the candidate anomalous traders detected by the weight-threshold method with the global threshold t = 7. Only the pink nodes are the candidate anomalous traders detected by our method. The weight of the unlabeled edges is 1.

Finally, we compare our method with the baseline method, i.e., the weight-threshold method. The weight-threshold method removes all the edges with a weight below a given threshold and then discards the isolated nodes. The remained nodes are taken as anomalous traders. This method has two evident shortcomings. Firstly, the threshold is difficult to be determined and it varies for different stocks. Secondly, for the normal traders having one edge with higher weight caused by random fluctuation, the weight threshold method will often misclassifies them into anomalous traders because of the absence of statistical significance. As a comparison, Figure 7 shows the anomalous traders detected by our method and the weight-threshold method. We can see that if a node has only one edge whose weight is greater than the threshold, the node would be anomalous node by the weight-threshold method. For our method, a node is an anomalous node only if it has a high average weight. To clarify the difference between our method and the weight-threshold method, we discuss the two possible reasons for the occurrence of an edge with higher weight. In the first case, if an investor submits a big bid order and a seller submits many ask orders at the same time, the system will match these two investors for many times. In the second case, several investors collude with each other and submit many ask/bid orders at the same time, and the weights among them are high. The weight-threshold method is inclined to take the nodes in the first case as anomalous traders while our method prefers to take the nodes in the second case as anomalous traders. Thus, our method is more effective to detect the anomalous traders who collude with each other to manipulate the price of stocks.

## Discussion

In this paper, we have analyzed the investors' behavior in the stock market and proposed a simple but effective method to detect anomalous traders in the trading network. Motivated by the observation that the trading network of manipulated stock exhibits high correlation between strength and degree, we detected anomalous traders by checking the statistical significance of each node in terms of the ratio of strength to degree. Besides the effectiveness, our method possesses an additional advantage, i.e., our method is hard to be fooled by colluded anomalous traders.

The colluded anomalous traders always attempt to trade frequently among themselves and to create an artificial demand for the stock. Their fraudulent trading behaviors will leave a trace in the trading network, e.g., the high correlation between strength and degree. The colluded traders will be doomed to detect by our method. If they try to evade the detection of our method, they have to trade less frequently among themselves. Then, they will take a long time to raise the stock price at their desired level. As a result, it becomes hard to attract other investors and the manipulation fails.

As future work, we will devote to the modeling of trading behavior of colluded traders and provides some insights about the manipulation mechanism. In this way, we can detect the colluded traders much earlier than existing methods and thus improve the online surveillance systems run by Stock Exchange.

## Materials and Methods

The results we present are produced using the transaction data of 52 stocks traded on the Shanghai Stock Exchange and the Shenzhen Stock Exchange during the whole year 2004, and these transaction data consist of executed orders. The data set of the 52 stocks contains 16,156,038 transaction entries, which involve 4,146,765 unique trader accounts. Each entry consists of the date and time, the unique number for the transaction, the buyer id, the seller id, the volume and price. Among all these 52 stocks, eight stocks had been manipulated by some investors and these manipulated stocks are revealed by CSRC for trade-based manipulation. In addition, for four of the eight manipulated stocks, the manipulated periods persist through the whole year of 2004 and the manipulated periods for the other four stocks are from Jan 2004 to Sep 2004.
